# Supplementary figures and images for: A glycolysis-related gene pairs signature predicts prognosis in patients with hepatocellular carcinoma
Source: PeerJ. 2020 Sep 29;8:e9944. doi: 10.7717/peerj.9944 (PMC7531359; doi:10.7717/peerj.9944)

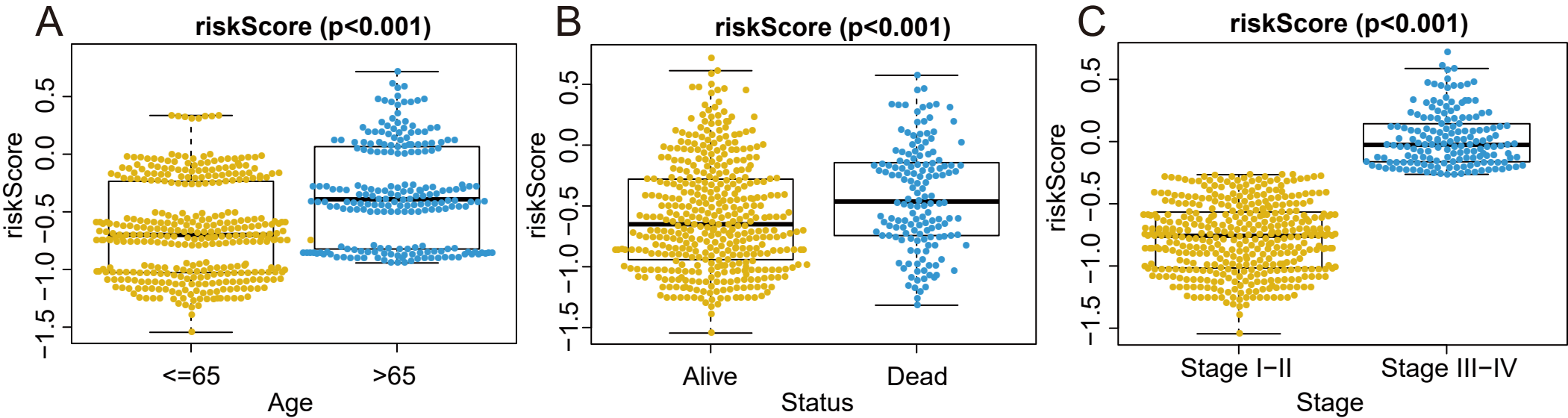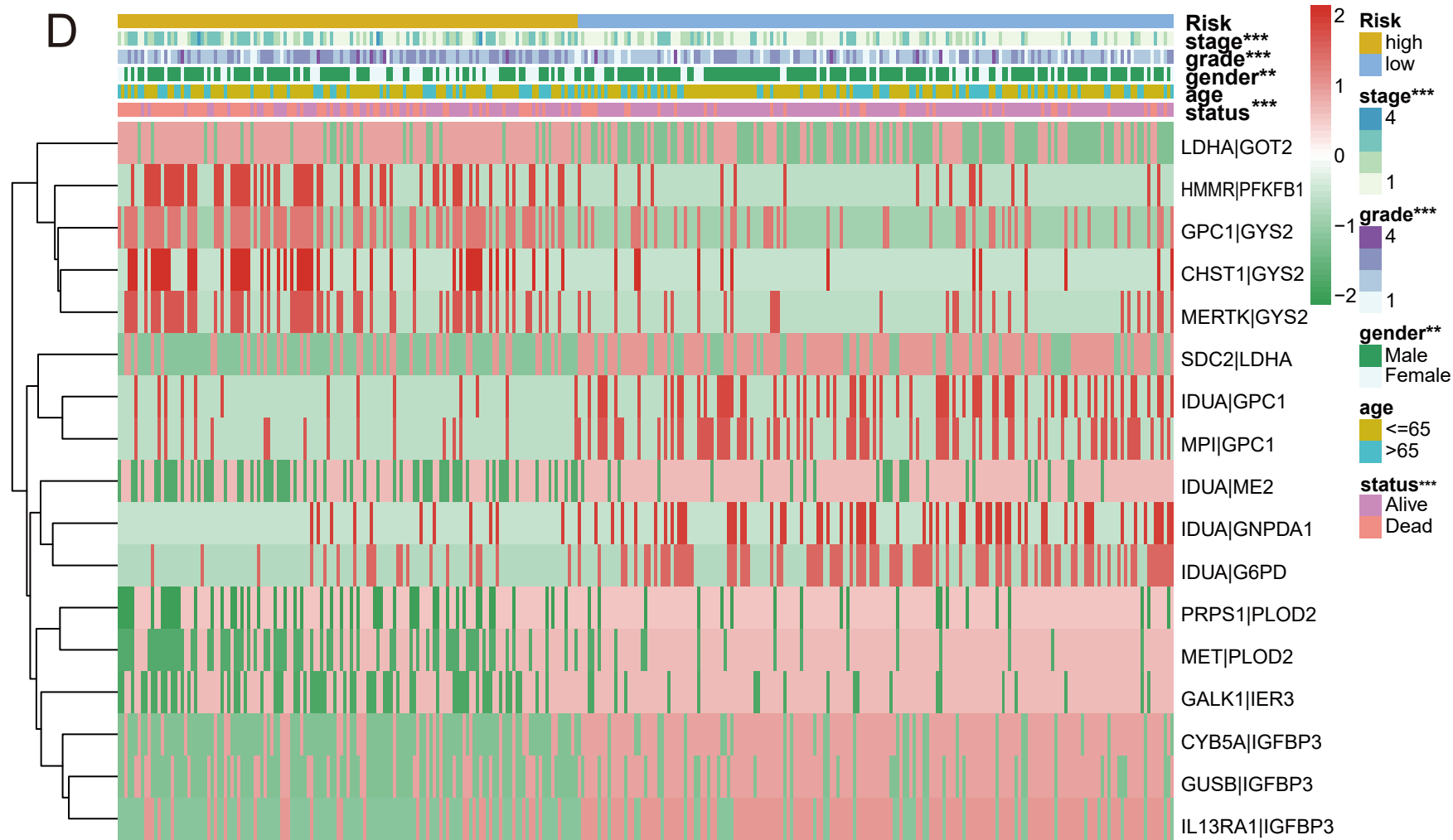

Supplement: Supplemental Information 1 — Statistical P values were significant at (A) age, (B) survival status, (C) TNM stage, (D) The heat map revealed the distribution of clinicopathological features and seventeen GRGPs expression contrasted between the low-risk and high-risk groups in TCGA dataset. [file peerj-08-9944-s001.pdf]
