# Supplementary material for: A glycolysis-related gene pairs signature predicts prognosis in patients with hepatocellular carcinoma
Source: PeerJ. 2020 Sep 29;8:e9944. doi: 10.7717/peerj.9944 (PMC7531359; doi:10.7717/peerj.9944)
Supplement: Supplemental Information 2 [file peerj-08-9944-s002.docx]

| NAME | SIZE | ES | NES | NOM  p-value | FDR  q-value | FWER  p-value | RANK  AT  MAX | LEADING EDGE |
| --- | --- | --- | --- | --- | --- | --- | --- | --- |
| HALLMARK_DNA_REPAIR | 150 | 0.74290407 | 2.1051989 | 0 | 0 | 0 | 10213 | tags=80%, list=18%, signal=98% |
| HALLMARK_MYC_TARGETS_V1 | 196 | 0.78198385 | 2.0081577 | 0 | 0.001023256 | 0.002 | 9215 | tags=87%, list=17%, signal=104% |
| HALLMARK_UNFOLDED_PROTEIN_RESPONSE | 110 | 0.7045137 | 1.9991038 | 0 | 0.001380583 | 0.003 | 10206 | tags=73%, list=18%, signal=89% |
| HALLMARK_E2F_TARGETS | 198 | 0.79814816 | 1.9078771 | 0 | 0.003618667 | 0.01 | 7766 | tags=87%, list=14%, signal=101% |
| HALLMARK_MTORC1_SIGNALING | 197 | 0.6264777 | 1.8855697 | 0 | 0.00417948 | 0.011 | 10660 | tags=59%, list=19%, signal=73% |
| HALLMARK_MYC_TARGETS_V2 | 58 | 0.7542948 | 1.8819315 | 0 | 0.0034829 | 0.011 | 5419 | tags=72%, list=10%, signal=80% |
| HALLMARK_PI3K_AKT_MTOR_SIGNALING | 105 | 0.65257156 | 1.8728967 | 0 | 0.004130642 | 0.014 | 10347 | tags=60%, list=19%, signal=74% |
| HALLMARK_G2M_CHECKPOINT | 196 | 0.7396588 | 1.8033252 | 0 | 0.009528583 | 0.034 | 10206 | tags=82%, list=18%, signal=100% |
| HALLMARK_WNT_BETA_CATENIN_SIGNALING | 42 | 0.6503722 | 1.7311162 | 0 | 0.016520774 | 0.087 | 14087 | tags=71%, list=25%, signal=96% |
| HALLMARK_SPERMATOGENESIS | 133 | 0.5181016 | 1.5940518 | 0.001449275 | 0.04777801 | 0.3 | 11387 | tags=38%, list=21%, signal=48% |
| HALLMARK_UV_RESPONSE_UP | 156 | 0.5121202 | 1.6920732 | 0.001564945 | 0.023265028 | 0.131 | 9450 | tags=43%, list=17%, signal=52% |
| HALLMARK_GLYCOLYSIS | 199 | 0.52456677 | 1.7434766 | 0.001567398 | 0.017257804 | 0.078 | 11802 | tags=49%, list=21%, signal=62% |
| HALLMARK_MITOTIC_SPINDLE | 198 | 0.6787043 | 1.7571924 | 0.003745318 | 0.01603378 | 0.063 | 10824 | tags=71%, list=20%, signal=88% |
| HALLMARK_HEME_METABOLISM | 195 | 0.5206248 | 1.6913157 | 0.0046875 | 0.0219273 | 0.131 | 16147 | tags=55%, list=29%, signal=77% |
| HALLMARK_APICAL_JUNCTION | 200 | 0.54012173 | 1.5835658 | 0.004769475 | 0.049213808 | 0.315 | 18158 | tags=62%, list=33%, signal=91% |
| HALLMARK_PROTEIN_SECRETION | 96 | 0.65859646 | 1.734368 | 0.005586592 | 0.0163078 | 0.082 | 13623 | tags=73%, list=25%, signal=97% |
| HALLMARK_P53_PATHWAY | 197 | 0.4941714 | 1.5943909 | 0.008156607 | 0.050467514 | 0.299 | 13771 | tags=51%, list=25%, signal=67% |
| HALLMARK_REACTIVE_OXYGEN_SPECIES_PATHWAY | 49 | 0.57559043 | 1.6564646 | 0.011764706 | 0.030708017 | 0.19 | 11810 | tags=57%, list=21%, signal=73% |
| HALLMARK_OXIDATIVE_PHOSPHORYLATION | 200 | 0.59916073 | 1.7198277 | 0.023214286 | 0.017123148 | 0.097 | 9655 | tags=58%, list=17%, signal=71% |
| HALLMARK_APOPTOSIS | 160 | 0.46422422 | 1.4599746 | 0.042158514 | 0.11908288 | 0.518 | 11279 | tags=39%, list=20%, signal=49% |
