# Supplementary material for: A glycolysis-related gene pairs signature predicts prognosis in patients with hepatocellular carcinoma
Source: PeerJ. 2020 Sep 29;8:e9944. doi: 10.7717/peerj.9944 (PMC7531359; doi:10.7717/peerj.9944)
Supplement: Supplemental Information 3 [file peerj-08-9944-s003.docx]

| Gene1 | Gene2 | HR | HR.95L | HR.95H | p-value |
| --- | --- | --- | --- | --- | --- |
| COG2 | PFKP | 0.521 | 0.364 | 0.746 | <0.001 |
| IDUA | GNPDA1 | 0.380 | 0.221 | 0.654 | <0.001 |
| IDUA | ME2 | 0.521 | 0.363 | 0.748 | <0.001 |
| IDUA | G6PD | 0.440 | 0.289 | 0.670 | <0.001 |
| IDUA | GPC1 | 0.378 | 0.220 | 0.650 | <0.001 |
| HMMR | PFKFB1 | 2.174 | 1.483 | 3.185 | <0.001 |
| KIF2A | PFKFB1 | 1.955 | 1.317 | 2.902 | 0.001 |
| MPI | GPC1 | 0.384 | 0.230 | 0.642 | <0.001 |
| SDC2 | LDHA | 0.447 | 0.307 | 0.652 | <0.001 |
| ENO1 | SOD1 | 1.892 | 1.303 | 2.747 | 0.001 |
| ME2 | PFKFB1 | 2.065 | 1.403 | 3.039 | <0.001 |
| ME2 | GYS2 | 1.874 | 1.290 | 2.722 | 0.001 |
| PRPS1 | PLOD2 | 0.497 | 0.336 | 0.734 | <0.001 |
| GALK1 | IER3 | 0.451 | 0.309 | 0.658 | <0.001 |
| GALK1 | IGFBP3 | 0.512 | 0.357 | 0.735 | <0.001 |
| CHST1 | GYS2 | 2.079 | 1.391 | 3.105 | <0.001 |
| MET | PLOD2 | 0.388 | 0.268 | 0.562 | <0.001 |
| PLOD2 | ALDH7A1 | 2.127 | 1.433 | 3.158 | <0.001 |
| GUSB | IGFBP3 | 0.459 | 0.319 | 0.662 | <0.001 |
| CHPF | IGFBP3 | 0.531 | 0.366 | 0.771 | 0.001 |
| PFKP | PFKFB1 | 1.976 | 1.353 | 2.885 | <0.001 |
| MERTK | GYS2 | 1.871 | 1.294 | 2.703 | 0.001 |
| IDH1 | IGFBP3 | 0.527 | 0.368 | 0.755 | <0.001 |
| AKR1A1 | IGFBP3 | 0.526 | 0.369 | 0.750 | <0.001 |
| PDK3 | GYS2 | 2.011 | 1.366 | 2.960 | <0.001 |
| GPC1 | GYS2 | 1.831 | 1.284 | 2.611 | 0.001 |
| ALDH7A1 | IER3 | 0.483 | 0.337 | 0.693 | <0.001 |
| IER3 | SLC37A4 | 1.840 | 1.284 | 2.637 | 0.001 |
| IL13RA1 | IGFBP3 | 0.421 | 0.290 | 0.610 | <0.001 |
| LDHA | GOT2 | 2.267 | 1.529 | 3.362 | <0.001 |
| SLC37A4 | IGFBP3 | 0.416 | 0.276 | 0.626 | <0.001 |
| PC | IGFBP3 | 0.446 | 0.308 | 0.645 | <0.001 |
| ALDH9A1 | IGFBP3 | 0.427 | 0.297 | 0.613 | <0.001 |
| GOT2 | IGFBP3 | 0.447 | 0.310 | 0.644 | <0.001 |
| CYB5A | IGFBP3 | 0.376 | 0.260 | 0.543 | <0.001 |
