# Supplementary material for: A glycolysis-related gene pairs signature predicts prognosis in patients with hepatocellular carcinoma
Source: PeerJ. 2020 Sep 29;8:e9944. doi: 10.7717/peerj.9944 (PMC7531359; doi:10.7717/peerj.9944)
Supplement: Supplemental Information 4 [file peerj-08-9944-s004.docx]

| Variable | B | SE | z | HR | HR.95L | HR.95H | *P* value |
| --- | --- | --- | --- | --- | --- | --- | --- |
| age | 0.005 | 0.007 | 0.705 | 1.005 | 0.991 | 1.020 | 0.481 |
| gender | -0.278 | 0.199 | -1.398 | 0.758 | 0.513 | 1.118 | 0.162 |
| grade | 0.114 | 0.133 | 0.864 | 1.121 | 0.865 | 1.454 | 0.388 |
| stage | 0.592 | 0.108 | 5.478 | 1.808 | 1.463 | 2.234 | <0.001 |
| Risk Score | 1.255 | 0.151 | 8.293 | 3.508 | 2.608 | 4.720 | <0.001 |
