# Supplementary material for: A glycolysis-related gene pairs signature predicts prognosis in patients with hepatocellular carcinoma
Source: PeerJ. 2020 Sep 29;8:e9944. doi: 10.7717/peerj.9944 (PMC7531359; doi:10.7717/peerj.9944)
Supplement: Supplemental Information 5 [file peerj-08-9944-s005.docx]

| Variable | B | SE | z | HR | HR.95L | HR.95H | *P* value |
| --- | --- | --- | --- | --- | --- | --- | --- |
| Gender | -0.005 | 0.004 | -1.305 | 0.995 | 0.988 | 1.002 | 0.192 |
| Age | -0.002 | 0.003 | -0.691 | 0.998 | 0.992 | 1.004 | 0.489 |
| Stage | 0.494 | 0.093 | 5.309 | 1.638 | 1.365 | 1.965 | <0.001 |
| Risk Score | 0.979 | 0.182 | 5.374 | 2.661 | 1.862 | 3.803 | <0.001 |
