# Supplementary material for: A glycolysis-related gene pairs signature predicts prognosis in patients with hepatocellular carcinoma
Source: PeerJ. 2020 Sep 29;8:e9944. doi: 10.7717/peerj.9944 (PMC7531359; doi:10.7717/peerj.9944)
Supplement: Supplemental Information 6 [file peerj-08-9944-s006.docx]

| Variable | B | SE | z | HR | HR.95L | HR.95H | *P* value |
| --- | --- | --- | --- | --- | --- | --- | --- |
| Gender | -0.026 | 0.007 | -3.689 | 0.974 | 0.961 | 0.988 | <0.001 |
| Age | -0.020 | 0.007 | -2.794 | 0.980 | 0.967 | 0.994 | 0.005 |
| Stage | 0.560 | 0.101 | 5.557 | 1.751 | 1.437 | 2.133 | <0.001 |
| Risk Score | 0.943 | 0.206 | 4.575 | 2.567 | 1.714 | 3.844 | <0.001 |
